# Pneumothorax: A rare presentation of pulmonary mycetoma

**DOI:** 10.4103/1817-1737.36554

**Published:** 2007

**Authors:** Prem Parkash Gupta, Sanjay Fotedar, Dipti Agarwal, Sarita Magu, Kuldeep Saini

**Affiliations:** *Department of Respiratory Medicine, Postgraduate Institute of Medical Sciences, Rohtak, India*; * *Department of Physiology and Postgraduate Institute of Medical Sciences, Rohtak, India*; ** *Department of Radiodiagnosis, Postgraduate Institute of Medical Sciences, Rohtak, India*

**Keywords:** Invasive fungal infection, mycetoma, pneumothorax

## Abstract

Pneumothorax due to mycetoma is extremely rare and has been described only in patients undergoing intensive cytotoxic therapy for hematologic malignancies. A non-immunocompromised subject presenting with pneumothorax due to rupture of the mycetoma into the pleural cavity is being described here.

*Aspergillus* infection in the lung is known to lead to obstructive bronchopulmonary aspergillosis, mucoid impaction of the airways, tracheobronchitis, ulcerative bronchitis, pseudomembranous tracheobronchitis, invasive aspergillosis or aspergilloma. Old tuberculosis cavities remain the most frequent underlying sites and were recorded in up to 70% of cases.[1] Out of various complications, massive hemoptysis is the most fearsome that necessitates an early surgical intervention. Pneumothorax is not identified as a common complication of ruptured mycetoma.

## Case Report

A 22-year-old male, farmer by occupation, presented with acute onset of breathlessness, chest pain over the right side of chest, moderate- to high-grade fever and dry cough - all for duration of 2 days. There was no history of diabetes, hypertension or any other significant disease/ disorder. The patient had a past history of smear-positive pulmonary tuberculosis 3 years back, for which he was advised antituberculosis drugs (ATT). He started the treatment but discontinued it after 2 months of his own as his symptoms were improved. Again, after a month, he developed the pulmonary symptoms, for which he was prescribed WHO Category-II regimen, thrice weekly for 8 months. This regimen included streptomycin, isoniazid, rifampicin, pyrazinamide and ethambutol for the initial 2 months; isoniazid, rifampicin, pyrazinamide and ethambutol for the next 1 month, after which pyrazinamide was stopped and the remaining three medicines were continued for 5 months. After completion of treatment, he was declared cured of pulmonary tuberculosis though he continued to have a thin-walled cavity.

**Figure 1:**
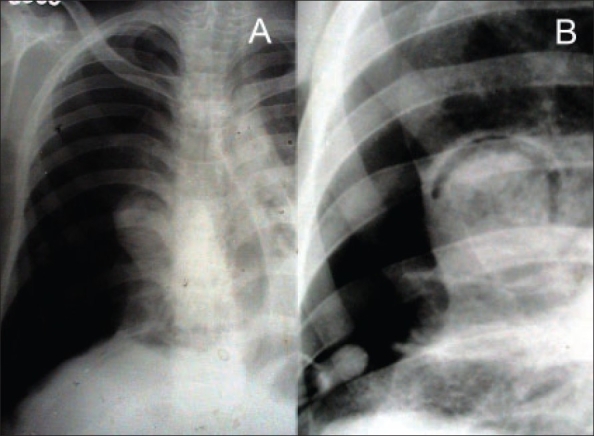
Chest roentgenogram PA view showing the right pneumothorax and the mycetoma with air-crescent sign; the tip of intercostal drain is also visible near the base.

**Figure 2:**
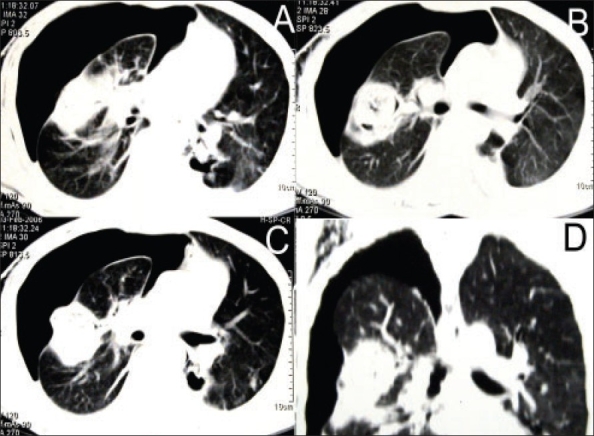
Computed tomogram of the thorax showing the various views of the mycetoma with rupture into the pleural cavity leading to pneumothorax.

He was of an average built and had good nutrition but was with apparent signs of respiratory distress. The movement of the thoracic cage was decreased over the right side with signs of increased lung volume. The right side was hyper-resonant all over the chest and the breath sounds were decreased. The patient underwent diagnostic work-up; his serum was nonreactive for HIV antigens (ELISA). The chest roentgenograms showed right-sided pneumothorax with a subpleural cavity containing a rounded opacity with the presence of air-crescent sign that was suggestive of mycetoma. Immediately, intercostal drainage under water seal was placed [Figure 1] and he was given supportive treatment. The computed tomography confirmed the diagnosis of mycetoma and that further revealed the rupture of the subpleural fungus ball, leading to pneumothorax [Figure 2]. Fiber-optic bronchoscopy revealed patent central airways. The bronchial secretions and the pleural aspirate showed *Aspergillus fumigatus*. He was also given anti-fungal treatment, itraconazole 200 mg daily, along with other supportive treatment. The patient responded to the management. He was under consideration for the surgical removal of the mycetoma; however, on the fifth day he had massive hemoptysis, all of a sudden and he expired.

## Discussion

*Aspergillus* is a ubiquitous fungus found in soil, water and decaying matter. It is known to cause a wide variety of respiratory conditions including allergic bronchopulmonary aspergillosis, parenchymal invasion and mycetoma. Mycetomas have a predisposition to occur in preexisting pulmonary cavities. *Aspergillus fumigatus* is the most common saprophytic species of *Aspergillus*, leading to aspergilloma.[2] In non-immunocompromised population, mycetomas have been reported to occur in 10-15% of patients with cavitating lung diseases.[3] In regions where the incidence of tuberculosis is on decline, mycetomas are increasingly identified in advanced sarcoidosis, pneumonoconioses, bullous emphysema, bronchiectasis, lung abscess, neoplasms and pulmonary infarcts.[4] Invasive aspergillus parenchymal infection is reported to affect 5-8% of solid-organ transplant recipients, with a 50-100% mortality.[5] In HIV seropositve patients, *P. carinii* pneumonia is a major risk factor for pulmonary aspergilloma.[6]

Patients with mycetoma are often asymptomatic. Amongst symptomatic, the most common symptoms include cough and hemoptysis. The course of disease is highly variable and patients are usually kept under observation without therapy unless they develop hemoptysis.[7] Massive hemoptysis necessitates surgical intervention with resection of the affected lung or arterial embolization in the patient who is not suitable for surgery. The antifungal drugs - itraconazole, voriconazole or amphotericin B - are used if complete surgical removal is not possible or if the *Aspergillus* infection has expanded beyond the aspergilloma.[8] A combination of antifungal and antiretroviral therapy has been shown to improve the clinical outcome in HIV-infected patients with pulmonary mycetoma.[6]

Pneumothorax due to rupture of a mycetoma into the pleural space in patients who are not otherwise immunocompromised, to the best of our knowledge, has not been reported in recent medical literature. Pneumothorax has been described in patients with pulmonary mycetoma undergoing intensive cytotoxic therapy for hematologic malignancies.[9] In the present case, the patient was not immunocompromised but still developed pneumothorax as a complication of the rupture of a mycetoma into the pleural space. It is significant to identify the link, as both pneumothorax and hemoptysis represent the clinical expression of a more destructive course of invasive fungal diseases and require aggressive medical and/or surgical management.
